# Serum CA125 is a novel predictive marker for pancreatic cancer metastasis and correlates with the metastasis-associated burden

**DOI:** 10.18632/oncotarget.6819

**Published:** 2016-01-05

**Authors:** Liang Liu, Hua-Xiang Xu, Wen-Quan Wang, Chun-Tao Wu, Jin-Feng Xiang, Chen Liu, Jiang Long, Jin Xu, De-Liang Fu, Quan-Xing Ni, Courtney W. Houchen, Russell G. Postier, Min Li, Xian-Jun Yu

**Affiliations:** ^1^ Department of Pancreatic Surgery, Fudan University, Shanghai Cancer Center, Shanghai 20032, P.R. China; ^2^ Pancreatic Cancer Institute, Fudan University, Shanghai 200032, P.R. China; ^3^ Department of Oncology, Shanghai Medical College, Fudan University, Shanghai 200032, P.R. China; ^4^ Department of Pancreatic Surgery, Huashan Hospital, Shanghai 200040, P.R. China; ^5^ Department of Medicine, The University of Oklahoma Health Sciences Center, Oklahoma City, OK 73104, USA; ^6^ Department of Surgery, The University of Oklahoma Health Sciences Center, Oklahoma City, OK 73104, USA

**Keywords:** serum CA125, metastasis, prognosis, pancreatic cancer

## Abstract

This study evaluated potential of serum tumor markers to predict the incidence and intensity of pancreatic cancer metastasis as well as patient survival. Retrospective records from 905 patients and prospective data from 142 patients were collected from two high-volume institutions. The levels of eight serum tumor markers (CA19-9, CEA, CA242, CA72-4, CA50, CA125, CA153, and AFP) commonly used in gastroenterological cancer were analyzed in all stages of pancreatic cancer. Serum CA125 levels were the most strongly associated with pancreatic cancer metastasis and were higher in patients with metastatic disease than those without. CA125 levels increased with increasing metastasis to lymph nodes and distant organs, especially the liver. High baseline CA125 levels predicted early distant metastasis after pancreatectomy and were associated with the presence of occult metastasis before surgery. An optimal CA125 cut-off value of 18.4 U/mL was identified; patients with baseline CA125 levels of 18.4 U/mL or higher had poor surgical outcomes. In addition, high serum CA125 levels coincided with the expression of a metastasis-associated gene signature and with alterations in “driver” gene expression involved in pancreatic cancer metastasis. CA125 may therefore be a promising, noninvasive, metastasis-associated biomarker for monitoring pancreatic cancer prognosis.

## INTRODUCTION

Pancreatic cancer is a lethal malignancy with high metastatic potential. Even small pancreatic cancers (less than 2 cm in diameter) metastasize, quickly resulting in death [1, 2]. Radical pancreatectomy is the only potential cure, but occult metastasis often diminishes its therapeutic effectiveness [3]. Both state-of-the-art preoperative imaging, including triple-phase helical computed tomography and positron emission tomography, and rigorous laparotomy exploration fail to detect occult metastasis before pancreatectomy [2, 3]. Identification of specific markers of micrometastasis when determining whether patients are candidates for pancreatectomy is therefore important for improving treatment.

Three genes that are frequently mutated in pancreatic cancer, TP53, CDKN2A/p16 and SMAD4/DPC4, modulate tumor metastasis and together comprise the “driver” gene signature [4, 5]. Additional metastasis-associated molecules, such as S100A2, also enhance the metastatic potential of pancreatic cancer. A recently identified group of 17 genes expressed in the bulk of primary tumors are predictive of the metastatic potential of most adenocarcinomas, including pancreatic cancer [6]. However, these biomarkers are difficult to measure due to limited sample availability and are typically examined postoperatively. Serologic biomarkers, particularly those that can be monitored easily in a relatively noninvasive and cost-effective manner, would be helpful for choosing treatment strategies.

Eight serological tumor markers (CA19-9, CEA, CA242, CA72-4, CA50, CA125, CA153, and AFP) are routinely used in clinical practice to make diagnoses, determine prognoses and monitor therapeutic responses in gastroenterological cancers. Among these, the most common and best-studied marker for pancreatic cancer is CA19-9 [2], which largely reflects tumor burden. We previously confirmed that serum levels of CA19-9 are associated with total tumor burden in pancreatic cancer [7], and do not specifically reflect the metastatic potential of the tumor, nor do they indicate the metastasis-associated burden. Additionally, CA19-9 levels do not change in some patients, even after complete resection of the tumor. This indicates the presence of occult unresectable disease, in particular micrometastasis, which developed before surgery [8, 9].

In this regards, we previously reported that two serum biomarkers, CEA and CA125, are preoperatively predictive of the absence of a postoperative decrease in CA19-9, and therefore suggest the presence of micrometastasis [10]. The predictive accuracy of CA125, determined by AUC analyses, was superior to that of CEA. Separately, we showed that CA125 was superior to CA19-9 for predicting resectability, which highlights the possible relationship between serum CA125 levels and occult unresectable disease in pancreatic cancer patients [11]. Here, we thoroughly examined the potential role of serum CA125 as a pretreatment biomarker for tumor metastasis-associated burden in pancreatic cancer.

## RESULTS

### High CA125 levels predict metastasis

**Figure 1:**
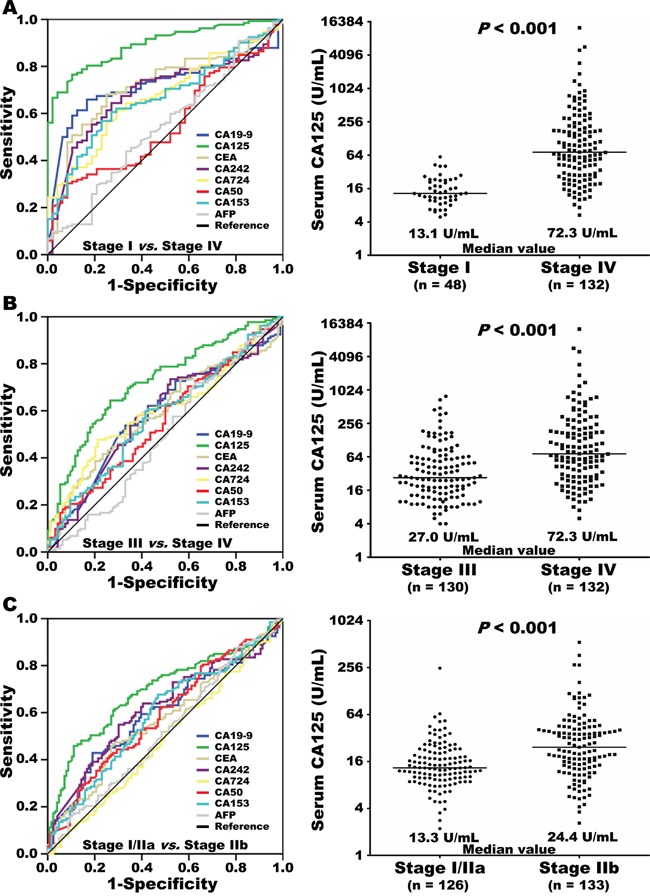
Eight serum tumor markers were included in receiver operating curve (ROC) analyses for prediction of metastasis, comparing stage I and stage IV pancreatic cancer **A.** Levels of baseline serum CA125 (log2 scale on the y-axis) in patients with stage I or IV disease (A). Eight serum tumor markers were further validated in ROC analyses for prediction of metastasis to distant organs in unresectable disease (stage III *vs.* stage IV) **B.** or to lymph nodes in radically resected disease (stage I/IIa *vs.* stage IIb) **C.** Levels of baseline serum CA125 (log2 scale) are plotted on the y-axis. The lines across the dot plots indicate median values.

Serum levels of CA19-9, CEA, CA242, CA72-4, CA50, CA125, CA153, and AFP were measured in 48 stage I and 132 stage IV pancreatic cancer patients. ROC curve analyses showed that increased CA125 levels were the best predictor of metastasis in these patients (AUC: 0.892, 95% CI [0.846, 0.938], *p*<0.001, Figure 1A). Patients with stage IV disease had higher serum CA125 levels than those with stage I disease (*p*<0.001). Furthermore, the ability of all eight markers to predict metastasis in subgroups with unresectable disease (stage III, locally advanced without distant metastasis *vs.* stage IV, with distant metastasis) and radically resected disease (stage I/IIa, without lymph node metastasis *vs.* stage IIb, with lymph node metastasis) was assessed. ROC curve analyses revealed that serum CA125 was the best predictor of metastasis to distant organs in the unresectable subgroup (AUC: 0.723, 95% CI [0.657, 0.789], *p*<0.001, Figure 1B), and to the lymph nodes in resected subgroup (AUC: 0.693, 95% CI [0.628, 0.758], *p*<0.001, Figure 1C). Patients with metastasis to either distant organs or lymph nodes had significantly higher serum CA125 levels than those without metastasis to the corresponding sites (*p*<0.001 for both, Figure 1B and 1C). Detailed data are shown in Table S1 and S2.

### High CA125 levels are associated with increased metastasis-associated tumor burden

**Figure 2:**
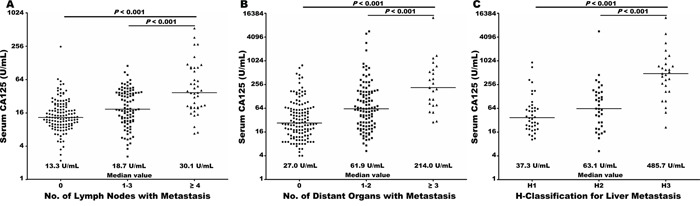
Baseline serum CA125 levels (log2 scale on the y-axis) were significantly elevated with increasing burden of metastasis to lymph nodes in radically resected disease **A.** and to distant organs in unresectable disease **B.** Baseline serum CA125 levels (log2 scale on the y axis) were significantly higher in patients with liver metastasis classified as H3 compared than those with liver metastasis classified as H2 classification or H1, respectively **C.** The lines across the dot plots indicate median values.

More in-depth subgroup analyses were performed to evaluate the relationship between baseline serum CA125 levels and metastasis to the lymph nodes and distant organs. In patients with resected disease (stage I–II), baseline serum CA125 levels increased as the number of metastatic lymph nodes increased (*r* = 0.304; *p*<0.001). CA125 levels were higher in patients with more than three metastatic lymph nodes than in those who had 1–3 or no metastatic lymph nodes (*p*<0.001 for both; Figure 2A). Similarly, in patients with unresectable pancreatic cancer (stage III–IV), CA125 levels were higher in those who had metastasis to more than three distant organs than in those who had 1–2 or no distant organ metastases (*p*<0.001 for both; Figure 2B). Especially in patients with liver metastasis, H-classification for the extent of metastasis was also evaluated by determining the size and number of metastatic foci. CA125 levels were higher in patients with H3 liver metastasis than in those with H2 or H1 classifications (*p*<0.001 for both; Figure 2C). These results indicate that baseline serum CA125 levels in pancreatic cancer reflect the extent of tumor dissemination to the lymph nodes, liver, and other metastatic sites.

### High CA125 levels are associated with early distant metastasis after pancreatectomy

We further explored whether CA125 levels predicted occult metastasis in the subgroup of patients who underwent pancreatectomy. ROC curve analyses showed that CA125 levels were associated with early postoperative metastasis of pancreatic cancer in distant organs (AUC: 0.720, 95% CI [0.646, 0.794], *p*<0.001). An optimal cut-off CA125 serum level of 18.4 U/mL was identified; patients with CA125 levels of 18.4 U/mL or higher had higher rates of early distant metastasis than those with lower levels (47/117 *vs.* 18/142, *p*<0.001, Table 1). The predictive sensitivity and specificity of CA125 levels for early metastasis in distant organs after pancreatectomy were 0.723 and 0.639, respectively. The cut-off value of 18.4 U/mL was also directly applied without re-estimation to an additional independent validation cohort. More of these patients with CA125 levels of 18.4 U/mL or higher had early postoperative metastasis than those with lower CA125 levels (55/165 *vs.* 6/108, *p*<0.001; Table 1). Previous studies showed that patients whose CA19-9 levels did not decrease after radical pancreatectomy were more likely to have had occult metastasis before surgery and to have early postoperative metastasis [8, 9]. Higher CA125 levels were associated with sustained postoperative CA19-9 levels in both the training (34/117 *vs.* 25/142, *p*=0.029) and validation (61/165 *vs.* 6/108, *p*<0.001) cohorts (Table 1).

**Table 1:** Relationship between clinicopathologic features and baseline serum CA125 Levels

Features	Training Cohort (n = 259)			Validation Cohort (n = 273)		
	Baseline Serum CA125 Levels		*P*	Baseline Serum CA125 Levels		*P*
	Negative (*n* = 142)	Positive (*n* = 117)		Negative (*n* = 108)	Positive (*n* = 165)	
**Age (years)**			0.073			0.217
< 62	63	65		58	76	
≥ 62	79	52		50	89	
**Gender**			0.536			0.324
Male	82	72		68	94	
Female	60	45		40	71	
**Tumor location**			0.777			0.445
Head	85	68		83	120	
Body/tail	57	49		25	45	
**Tumor size (cm)**			0.001			0.026
≤ 4.0	85	46		62	72	
> 4.0	57	71		46	93	
**Lymph node metastasis**			< 0.001			0.001
Yes	52	81		48	108	
No	90	36		60	57	
**Differentiation**			0.093			0.197
Well/Moderate	44	48		75	102	
Poor	98	69		33	63	
**Neural invasion**			0.388			0.268
Yes	117	101		69	116	
No	25	16		39	49	
**Vascular invasion**			0.237			0.822
Yes	31	33		32	51	
No	111	84		76	114	
**TNM stage**			< 0.001			0.001
I	33	15		32	22	
IIA	57	21		28	35	
IIB	52	81		48	108	
**Chemotherapy**			0.361			0.912
Any	118	92		74	112	
No	24	25		34	53	
**Chemoradiotherapy**			0.274			0.390
Any	40	26		13	26	
No	102	91		95	139	
**CA19-9 non-decrease**			0.029			< 0.001
Yes	25	34		6	61	
No	117	83		102	104	
**Early distant metastasis**			< 0.001			< 0.001
Yes	18	47		6	55	
No	124	70		102	110	

Note: 18.4 U/mL was identified by ROC curve analysis as the cut-off value for positive/negative baseline serum CA125. Early distant metastasis is defined as the recurrence in distant organs within 6 months after surgery

To best mimic clinical practice, we expanded the validation cohort to include more patients from the retrospective database regardless of baseline CA19-9 and preoperative bilirubin levels. The expanded analyses still showed that preoperative CA125 levels of 18.4 U/mL or higher predicted postoperative early distant metastasis in the expanded validation cohort, AUC: 0.671, 95% CI [0.612, 0.731], *p*<0.001. Patients with baseline CA125 levels of at least 18.4 U/mL were more likely to experience distant metastasis within 6 months of pancreatectomy (74/232 *vs.* 11/152, *p*<0.001). Analyses of the prospective pancreatectomy database from our institution also confirmed these results. Of the 142 patients in that database who met the expanded inclusion criteria described above, the 83 patients with baseline CA125 levels of 18.4 U/mL or higher were more likely to experience early distant metastasis postoperatively than those with lower CA125 levels (30/83 *vs.* 7/59, *p*=0.001).

### High CA125 levels, and sustained levels postoperatively, predict poor OS and RFS

**Figure 3:**
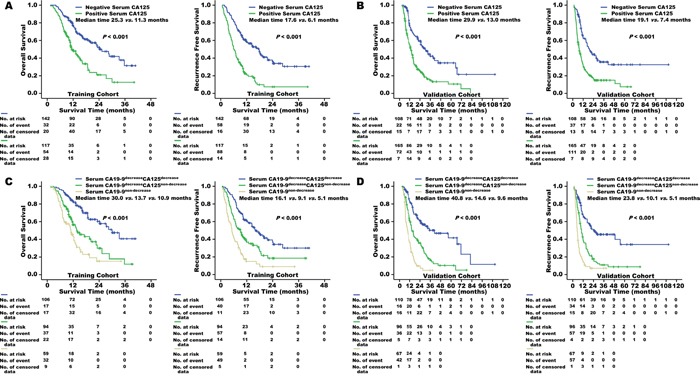
Overall survival and recurrence-free survival of patients in the training cohort **A.** and the validation cohort **B.** were stratified by positive/negative baseline serum CA125 levels. The cut-off value of serum CA125 for predicting pancreatic cancer metastasis was identified as 18.4 U/mL. In patients with a decrease in postoperative serum CA19-9, postoperative serum CA125 decrease/non-decrease further distinguished overall survival and recurrence-free survival in both the training cohort **C.** and the validation cohort **D.**

As shown in Figure 3A, resected patients with baseline CA125 levels of 18.4 U/mL or higher had shorter median overall survival (OS) (11.3 *vs.* 25.3 months, *p*<0.001) and relapse-free survival (RFS) (6.1 *vs.* 17.6 months, *p*<0.001) than did those with lower levels in the training cohort. Multivariate Cox proportional hazards analyses that adjusted for clinicopathological features demonstrated that CA125 levels of at least 18.4 U/mL were an independent risk factor for both OS (HR: 1.804, 95% CI [1.222, 2.662], *p*=0.003) and RFS (HR: 2.158, 95% CI [1.530, 3.043], *p*<0.001; Table 2) in these patients. Similar results were seen in the validation cohort (Table 2 and Figure 3B).

**Table 2:** Univariate and multivariate analyses for OS and RFS in pancreatic cancer after radical resection

Features	OS						RFS					
	Univariate Analyses			Multivariate Analyses			Univariate Analyses			Multivariate Analyses		
	HR	95 % CI	*P*	HR	95 % CI	*P*	HR	95 % CI	*P*	HR	95 % CI	*P*
***Training Cohort (n = 259)***												
**Age** (< 62 *vs.* ≥ 62 years)	1.065	[0.755, 1.503]	0.719	0.977	[0.677, 1.410]	0.901	0.869	[0.645, 1.169]	0.353	0.869	[0.632, 1.194]	0.385
**Gender** (female *vs.* male)	1.000	[0.704, 1.420]	1.000	0.968	[0.668, 1.403]	0.865	1.197	[0.881, 1.625]	0.246	1.149	[0.833, 1.585]	0.398
**Location** (head *vs.* body/tail)	0.896	[0.630, 1.275]	0.543	0.855	[0.582, 1.255]	0.423	1.018	[0.753, 1.376]	0.906	1.038	[0.746, 1.442]	0.826
**Serum CA19-9** (≤ 37.0 *vs.* > 37.0 U/mL)	1.977	[1.271, 3.139]	0.003	1.903	[1.195, 3.031]	0.007	2.171	[1.476, 3.194]	< 0.001	1.954	[1.309, 2.917]	0.001
**Serum CA125** (≤ 18.4 *vs.* > 18.4 U/mL)	2.431	[1.708, 3.460]	< 0.001	1.804	[1.222, 2.662]	0.003	2.841	[2.086, 3.871]	< 0.001	2.158	[1.530, 3.043]	< 0.001
**Tumor size** (≤ 4.0 cm *vs.* > 4.0 cm)	1.779	[1.249, 2.532]	0.001	1.790	[1.219, 2.628]	0.003	1.534	[1.137, 2.069]	0.005	1.385	[0.998, 1.924]	0.052
**Lymph node metastasis** (no *vs.* yes)	2.667	[1.856, 3.883]	< 0.001	-	-	-	2.363	[1.740, 3.210]	< 0.001	-	-	-
**Differentiation** (I/II *vs.* III)	1.645	[1.157, 2.341]	0.006	1.622	[1.120, 2.348]	0.010	1.586	[1.168, 2.153]	0.003	1.517	[1.099, 2.094]	0.011
**Neural invasion** (no *vs.* yes)	1.279	[0.786, 2.083]	0.322	1.133	[0.688, 1.865]	0.624	1.308	[0.856, 1.999]	0.215	1.032	[0.669, 1.592]	0.886
**Microvascular invasion** (no *vs.* yes)	1.738	[1.192, 2.534]	0.004	1.560	[1.047, 2.324]	0.029	1.618	[1.160, 2.257]	0.005	1.354	[0.947, 1.938]	0.097
**TNM stage** (I *vs.* IIA *vs.* IIB)	2.106	[1.607, 2.759]	< 0.001	2.220	[1.657, 2.975]	< 0.001	1.869	[1.502, 2.326]	< 0.001	1.738	[1.376, 2.194]	< 0.001
**Chemotherapy** (no *vs.* yes)	0.279	[0.190, 0.410]	< 0.001	0.164	[0.105, 0.257]	< 0.001	0.472	[0.332, 0.670]	< 0.001	0.283	[0.190, 0.423]	< 0.001
**Chemoradiotherapy** (no *vs.* yes)	0.896	[0.607, 1.322]	0.579	1.086	[0.693, 1.703]	0.719	1.280	[0.928, 1.765]	0.133	1.520	[1.057, 2.184]	0.024
***Validation Cohort (n = 273)***												
**Age** (< 62 *vs.* ≥ 62 years)	1.014	[0.759, 1.355]	0.924	0.978	[0.725, 1.321]	0.887	1.062	[0.802, 1.405]	0.676	1.003	[0.752, 1.338]	0.984
**Gender** (female *vs.* male)	0.962	[0.716, 1.291]	0.796	0.984	[0.726, 1.335]	0.919	1.017	[0.765, 1.352]	0.909	0.994	[0.742, 1.332]	0.968
**Location** (head *vs.* body/tail)	1.018	[0.722, 1.436]	0.917	0.921	[0.640, 1.326]	0.659	1.034	[0.748, 1.429]	0.840	1.023	[0.726, 1.442]	0.896
**Serum CA19-9** (≤ 37.0 *vs.* > 37.0 U/mL)	1.828	[1.289, 2.594]	0.001	1.610	[1.124, 2.304]	0.009	1.864	[1.326, 2.620]	< 0.001	1.751	[1.233, 2.489]	0.002
**Serum CA125** (≤ 18.4 *vs.* > 18.4 U/mL)	2.451	[1.777, 3.380]	< 0.001	2.135	[1.526, 2.988]	< 0.001	2.392	[1.762, 3.246]	< 0.001	2.121	[1.541, 2.919]	< 0.001
**Tumor size** (≤ 4.0 cm *vs.* > 4.0 cm)	1.683	[1.253, 2.261]	0.001	1.547	[1.125, 2.129]	0.007	1.606	[1.210, 2.131]	0.001	1.403	[1.037, 1.889]	0.028
**Lymph node metastasis** (no *vs.* yes)	2.204	[1.606, 3.024]	< 0.001	-	-	-	2.154	[1.593, 2.915]	< 0.001	-	-	-
**Differentiation** (I/II *vs.* III)	1.523	[1.128, 2.055]	0.006	1.031	[0.751, 1.415]	0.851	1.433	[1.073, 1.914]	0.015	1.045	[0.773, 1.412]	0.776
**Neural invasion** (no *vs.* yes)	1.217	[0.889, 1.666]	0.221	1.226	[0.883, 1.701]	0.224	1.226	[0.904, 1.662]	0.189	1.224	[0.895, 1.673]	0.206
**Microvascular invasion** (no *vs.* yes)	1.630	[1.198, 2.218]	0.0072	1.553	[1.123, 2.147]	0.008	1.479	[1.097, 1.994]	0.010	1.382	[1.009, 1.893]	0.044
**TNM stage** (I *vs.* IIA *vs.* IIB)	1.718	[1.398, 2.111]	< 0.001	1.716	[1.363, 2.160]	< 0.001	1.699	[1.391, 2.075]	< 0.001	1.668	[1.332, 2.089]	< 0.001
**Chemotherapy** (no *vs.* yes)	0.662	[0.485, 0.902]	0.009	0.466	[0.331, 0.655]	< 0.001	0.747	[0.553, 1.009]	0.058	0.518	[0.372, 0.720]	< 0.001
**Chemoradiotherapy** (no *vs.* yes)	1.230	[0.839, 1.803]	0.289	1.180	[0.799, 1.744]	0.406	1.582	[1.105, 2.266]	0.012	1.473	[1.019, 2.128]	0.039

Note: All factors except lymph node metastasis were adopted in multivariate analyses because TNM stage was classified based on lymph node metastasis and was influenced by lymph node metastasis in multivariate analyses

Serum CA125 levels decreased in some resected patients after pancreatectomy (120/259 for the training cohort and 129/273 for the validation cohort). This decrease was associated with longer median OS (28.4 *vs.* 12.3 months, *p*<0.001 for the training cohort; 26.7 *vs.* 11.7 months, *p*<0.001 for the validation cohort) and RFS (15.1 *vs.* 6.8 months, *p*<0.001 for the training cohort; 19.5 *vs.* 7.6 months, *p*<0.001 for the validation cohort) in these patients. Postoperative decreases in CA19-9 levels were even more common. However, among patients with postoperative decreases in serum CA19-9 levels, nearly half (47.0% (94/200) in the training cohort and 46.6% (96/206) in the validation cohort) had sustained CA125 levels. Sustained CA125 levels also predicted higher rates of early metastasis in distant organs (27/94 *vs.* 9/106, *p*<0.001 for the training cohort; 19/96 *vs.* 7/110, *p*<0.001 for the validation cohort) and shorter OS and RFS times in patients (Figure 3C and 3D).

### High CA125 levels correspond to a metastasis-associated gene signature

**Figure 4:**
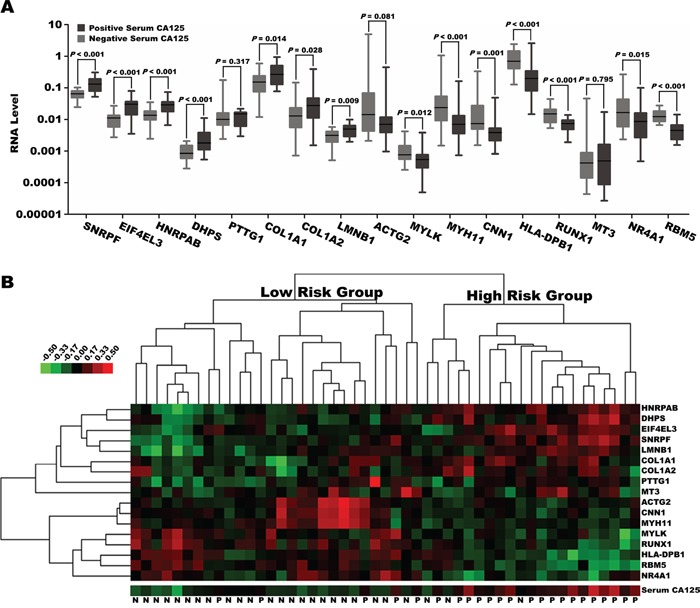
**A.** Expression of 17 genes (log10 scale on the y axis) included in the metastasis-associated gene signature was analyzed in patients with pancreatic cancer with positive or negative serum CA125 expression. The cut-off value of serum CA125 for predicting pancreatic cancer metastasis was identified as 18.4 U/mL. The lines across the dot plots indicate the median values. **B.** Two categories of patients classified by positive/negative baseline serum CA125 levels were largely consistent with two clusters based on hierarchical clustering of the metastasis-associated gene signature.

Of the 107 patients who underwent pancreatectomy, KRAS protein expression increased and CDKN2A/p16, TP53, and SMAD4/DPC4 protein expression decreased in 93 (86.9%), 41 (38.3%), 51 (47.7%), and 54 (50.5%) patients, respectively. CA125 levels did not differ between patients who were positive and negative for KRAS expression (Figure S1A), but were significantly higher in patients who did not express CDKN2A/p16, TP53, or SMAD4/DPC4 than in patients who did (Figure S1A). CA125 levels were higher still in patients with 3-4 additional altered genes than in those with 0-2 additional altered genes (*p*<0.001; Figure S1B and S1C). The expression of a well-established metastasis-associated gene signature, consisting of eight upregulated and nine downregulated genes, was also examined in an independent sample of 49 pancreatic cancer patients who underwent resection (Stage I/II). Seven of the eight upregulated metastatic genes (SNRPF, EIF4EL3, HNRPAB, DHPS, COL1A1, COL1A2, and LMNB1) were overexpressed in patients with CA125 levels of at least 18.4 U/mL. The expression of seven of the nine downregulated metastatic genes (MYLK, MYH11, CNN1, HLA-DPB1, RUNX1, NR4A1, and RBM5) decreased in patients with CA125 levels of at least 18.4 U/mL (Figure 4A). Hierarchical clustering revealed that dichotomized baseline serum CA125 values (cut-off value: 18.4 U/mL) reproduced the two clusters of pancreatic cancer patients identified using the 17 unique metastasis-associated genes (*p*<0.001; Figure 4B).

## DISCUSSION

Here we provide additional evidence that CA125 levels are predictive of metastasis. We found that high baseline CA125 levels were the best predictor of pancreatic cancer metastasis. CA125 levels were significantly elevated higher in patients with metastasis compared tothan those without. CA125 levels could predict the presence of lymph node metastasis in patients with resectable disease (stages IIa and IIb) and the presence of distant metastasis in patients with unresectable disease (stages III and IV). Second, more in-depth analysis of subgroups indicated that CA125 levels increased as the number of lymph nodes colonized by metastatic tumor cells increased. Furthermore, in patients with distant metastasis, increases in CA125 levels were more pronounced in those with extensive metastasis at multiple distant organs and in those with a heavy liver metastasis burden. This suggests that serum CA125 levels indicate not only metastatic potential, but also the extent of metastasis in pancreatic cancer patients. Third, patients with baseline serum CA125 levels of 18.4 U/mL or higher were more likely to have early postoperative recurrence in distant organs than those with lower CA125 levels. Even in some patients with exhibiting postoperative decreases in CA19-9, sustained CA125 levels were observed after surgery and coincided with higher rates of early distant metastasis and a poor prognosis. Finally, CA125 expression levels in pancreatic cancer tissues were positively correlated with serum levels, and expression was specific to tumor cells (Figure S2A-C). CA125 expression was higher in most metastatic lesions, including the lymph nodes and liver, than in matched primary tumors (Figure S2D). Additionally, high CA125 levels in serum positively correlated with genetic alterations in “driver” genes, and especially with co-alteration of *CDKN2A/p16*, *TP53*, and *SMAD4/DPC4*, in pancreatic cancer specimens. Separating patients into two groups based on CA125 levels (cut-off value: 18.4 U/mL) recreated two pancreatic cancer clusters previously identified using a well-known metastasis-associated gene signature for adenocarcinomas.

CA125 is the “classic” biomarker for ovarian cancer [12], but its diagnostic and prognostic value in pancreatic cancer patients is less studied. To our knowledge, the present data demonstrate for the first time the utility of CA125 levels for predicting metastasis-associated burden in pancreatic cancer. A reanalysis of longitudinal data from the United Kingdom Collaborative Trial of Ovarian Cancer Screening (UKCTOCS) [13], which is the largest reported ovarian cancer screening trial and included 154 women who subsequently developed pancreatic cancer, supported this role for CA125 levels. In that study, including serum CA125 measurements improved detection of preclinical pancreatic cancer based on CA19-9 levels, particularly in CA19-9-negative cases. We previously found that high baseline serum CA125 levels predicted the non-decrease of CA19-9 levels postoperatively and poor survival in the subgroup of patients with baseline CA19-9 levels of 1,000 U/mL or higher [10]. Here, most patients with high CA125 levels who underwent pancreatectomy had worse OS and RFS than those with low levels of CA125. Furthermore, the relationship between early postoperative metastasis and high CA125 levels was independent of CA19-9 levels. Analyses of the expanded retrospective database and another independent prospective database showed that most patients with high CA125 levels who underwent pancreatectomy experienced early postoperative metastasis at distant organs, regardless of other factors, including CA19-9 levels or preoperative hyperbilirubinemia. Together, these results suggest that serum CA125 levels may serve as a valuable clinical biomarker of occult disease in pancreatic cancer.

We also observed a perioperative change in CA125 levels in some pancreatic cancer patients who underwent pancreatectomy, as is the case with CA19-9. Serum CA125 and CA19-9 levels, whether preoperative or postoperative, independently predicted both OS and RFS in patients who underwent pancreatectomy (Table 3). However, while more than 80% of patients show decreases in CA19-9 levels after pancreatectomy [8, 9], a smaller proportion of patients showed postoperative decreases in CA125 levels (120/259 *vs.* 200/259 for CA19-9). It seems to mean that CA125 levels are less sensitive to primary tumor burden. Moreover, we found that nearly 50% of the patients in this study, who experienced a decrease in postoperative serum CA19-9 levels, still experienced an early distant metastasis and poor survival because they did not show a decrease in CA125 levels. Only a few patients who had a postoperative decrease in CA125 levels did not show a decrease in CA19-9 levels. In addition, a comparison with CA19-9 showed that preoperative high levels of serum CA19-9 predicted primary tumor staging at the T3 stage better than did high CA125, AUC: 0.578, P=0.049 for CA19-9, and AUC: 0.541, P=0.308 for CA125. And serum CA125 showed superiority to serum CA19-9 in predicting lymph node staging at the N1 stage, AUC: 0.693, P<0.001 for CA125, and AUC: 0.598, P=0.006 for CA19-9; difference between AUC: 0.094, P=0.024. These observations further suggest that CA125 levels play a unique role as a marker of pancreatic cancer metastasis and that CA125 is a better predictor of metastatic tumor burden than CA19-9. Thus, monitoring perioperative changes in CA125 levels, which are indicative of metastasis potential, might improve outcome predictions and treatment decisions when monitored alongside CA19-9 levels.

**Table 3:** Univariate and multivariate cox regression analyses of serum tumor markers for OS and RFS in shanghai cancer center

Features	OS						RFS					
	Univariate analyses			Multivariate Analyses			Univariate analyses			Multivariate Analyses		
	HR	95 %	*P*	HR	95 %	*P*	HR	95 %	*P*	HR	95 %	*P*
**Preoperative CA19-9** (≥ 37 vs.< 37 U/mL)	1.997	[1.271, 3.139]	0.003	1.811	[1.148-2.856]	0.011	2.171	[1.476, 3.194]	< 0.001	1.869	[1.262, 2.768]	0.002
**Preoperative CA125** (≥ 18.4 vs.< 18.4 U/mL)	2.431	[1.708, 3.460]	< 0.001	2.315	[1.620-3.308]	< 0.001	2.841	[2.086, 3.871]	< 0.001	2.454	[1.778, 3.387]	< 0.001
**Preoperative CEA** (≥ 5.2 vs.< 5.2 ng/mL)	1.587	[1.103, 2.285]	0.013			NS	1.766	[1.292, 2.412]	< 0.001	1.467	[1.065, 2.022]	0.019
**Preoperative CA242** (≥20 vs.< 20 U/mL)	1.795	[1.249, 2.580]	0.002			NS	1.882	[1.381, 2.566]	< 0.001			NS
**Preoperative CA724** (≥6.9 vs.< 6.9 U/mL)	0.988	[0.619, 1.576]	0.958			NS	1.271	[0.866, 1.866]	0.221			NS
**Preoperative CA50** (≥25 vs.< 25 U/mL)	1.530	[1.060, 2.207]	0.023			NS	1.382	[1.018, 1.877]	0.038			NS
**Preoperative CA153** (≥25 vs.< 25 U/mL)	1.114	[0.668, 1.858]	0.678			NS	1.202	[0.775, 1.865]	0.410			NS
**Preoperative AFP** (≥10 vs.< 10 ng/mL)	0.609	[0.248, 1.492]	0.278			NS	0.555	[0.260, 1.184]	0.127			NS
**Postoperative CA19-9** (non-decrease vs. decrease)	2.551	[1.756, 3.704]	< 0.001	2.008	[1.352-2.982]	0.001	2.568	[1.844, 3.576]	< 0.001	2.374	[1.677, 3.360]	< 0.001
**Postoperative CA125** (non-decrease vs. decrease)	2.579	[1.787, 3.720]	< 0.001	2.078	[1.419-3.041]	< 0.001	1.914	[1.411, 2.597]	< 0.001	1.600	[1.166, 2.195]	0.004
**Postoperative CEA** (non-decrease vs. decrease)	1.954	[1.378, 2.770]	< 0.001	1.467	[1.017-2.117]	0.040	1.618	[1.191, 2.199]	0.002			NS
**Postoperative CA242** (non-decrease vs. decrease)	1.685	[1.183, 2.400]	0.004			NS	1.403	[1.029, 1.914]	0.032			NS
**Postoperative CA724** (non-decrease vs. decrease)	1.431	[1.010, 2.029]	0.044			NS	1.392	[1.030, 1.880]	0.031			NS
**Postoperative CA50** (non-decrease vs. decrease)	1.822	[1.272, 2.608]	0.001			NS	1.594	[1.165, 2.183]	0.004			NS
**Postoperative CA153** (non-decrease vs. decrease)	1.562	[1.106, 2.206]	0.011			NS	1.384	[1.027, 1.864]	0.033			NS
**Postoperative AFP** (non-decrease vs. decrease)	1.159	[0.792, 1.698]	0.448			NS	1.010	[0.722, 1.415]	0.952			NS

Note: All eight serum markers were adopted in multivariate analyses with forward-stepwise features selection. NS: not significant

In sum, we have described a unique role for serum CA125 levels in pancreatic cancer diagnosis and treatment. CA125 levels specifically reflect the metastasis-associated burden of pancreatic cancer in patients with advanced disease, as well as the presence of occult metastasis in patients with clinically localized tumors. Incorporating routine analysis of serum CA125 levels in clinical examinations both before and after pancreatic cancer treatments may help to improve therapeutic decisions and patient survival.

## MATERIALS AND METHODS

### Patients and study design

Between January 2010 and December 2012, we retrospectively screened a total of 521 patients with pancreatic adenocarcinoma that was pathologically diagnosed at our institution (Shanghai Cancer Center, Table 4). The following exclusion criteria were applied: (a) incomplete clinicopathological and follow-up data, (b) any anti-tumor treatment before surgery, (c) serum CA19-9 levels persistently <5 U/mL, and (d) serum total bilirubin levels ≥2.0 mg/dL. The last two criteria were included to remove distractions from CA19-9, which is the “gold standard” to which newly discovered pancreatic cancer markers are compared. Patients meeting the criteria were divided into five groups according to the TNM staging criteria for pancreatic cancer in the 7th edition of the American Joint Committee on Cancer (AJCC) Cancer Staging Manual [14] as follows: (i) stage I (*n*=48), (ii) stage IIa (*n*=78), (iii) stage IIb (*n*=133), (iv) stage III (*n*=130), and (v) stage IV (*n*=132). We initially investigated the expression levels of all serum tumor markers that are currently applied in gastrointestinal cancer (AFP, CA19-9, CEA, CA242, CA72-4, CA50, CA125, and CA153) in a subset of serum specimens from the stage IV (*n*=132) and stage I groups (*n*=48). This was done to identify candidate biomarkers that are specifically associated with pancreatic cancer metastasis. Next, the ability of candidate markers to predict metastasis was confirmed in subgroups with similar primary tumor burdens. Patients with unresectable disease, including those assigned to stage III (*n*=130) and stage IV (*n*=132) groups due to distant metastasis, and patients who underwent resection, including those assigned to stage I/IIa (*n*=126) and stage IIb (*n*=133) groups based on lymph node metastasis, were included at this point in the study. All patients enrolled during the initial discovery of candidate markers were also included in this portion of the study and were assigned to the corresponding groups. Finally, we evaluated the predictive value of candidate markers for early distant metastasis and surgical prognosis in resected patients. Patients with stage I, IIa, and IIb disease who underwent pancreatectomy were included in the training cohort (*n*=259). A second independent group of 273 patients who underwent a similar operation between January 2003 and December 2012 at another high-volume center (Shanghai Huashan Hospital) were included as a validation cohort according to the same inclusion/exclusion criteria described above. In order to accurately assess all candidate markers and mimic clinical practice, patients with CA19-9 levels persistently <5 U/mL (*n*=23) and preoperative total bilirubin levels ≥2.0 mg/dL (*n*=88) who underwent radical pancreatectomy at this center were also included in validation analyses (Table S3). In addition, we retrospectively analyzed an independent prospective dataset that included 142 patients with pancreatic head carcinoma who underwent pancreaticoduodenectomy at our institution between November 2012 and December 2014 (ClinicalTrials.gov identifier: NCT01731821; Table S3). These data helped to validate the findings of this study, which otherwise lacked separation between data from pancreatic body and tail cancers.

**Table 4:** Clinicopathological features of patients with pancreatic cancer in training cohort and validation cohort

Features	Radically resected disease	Unresectable disease		Radically resected disease
	Stage I/II (Training cohort)	Stage III (no metastasis)	Stage IV (metastasis)	Stage I/II (Validation cohort)
	*n* = 259	*n* = 130	*n* = 132	*n* = 273
**Age** [years, median (range)]	62 (28 - 84)	65 (39 - 79)	60 (28 - 81)	62 (20 - 79)
**Gender** (male/female)	154/105	69/61	83/49	162/111
**Tumour location** (head/body, tail)	153/106	72/58	59/73	203/70
**Serum CA19-9** [U/mL, median (range)]	169.4 (5.1 - 17690.0)	341.3 (5.6 - 21430.0)	891.1 (6.2 - 25280.0)	146.3 (5.4 - 20740.0)
**Serum CA125** [U/mL, median (range)]	17.3 (2.2 - 539.7)	27.0 (4.0 - 786.2)	72.3 (5.2 - 12751.0)	24.2 (2.4 - 666.5)
**Serum CEA** [ng/mL, median (range)]	3.5 (0.4 - 406.9)	4.7 (0.8 - 444.1)	6.9 (0.7 - 951.0)	2.7 (0.3 - 256.0)
**Serum CA242** [U/mL, median (range)]	30.0 (0.1 - 238.1)	39.4 (0.1 - 216.0)	115.9 (0.1 - 316.0)	24.8 (0.1 - 298.0)
**Serum CA72-4** [U/mL, median (range)]	2.3 (0.7 - 1190.8)	3.0 (0.8 - 148.8)	5.0 (0.7 - 300.0)	3.0 (0.8 - 253.8)
**Serum CA50** [U/mL, median (range)]	35.3 (0.6 - 1120.9)	20.4 (1.5 - 1047.7)	23.5 (0.8 - 1126.0)	28.7 (0.4 - 380.0)
**Serum CA153** [U/mL, median (range)]	12.5 (4.4 - 230.1)	13.6 (4.1 - 163.6)	16.7 (4.7 - 300.0)	19.3 (5.2 - 57.4)
**Serum AFP** [ng/mL, median (range)]	3.0 (0.7 - 3000.0)	2.9 (0.9 - 58.2)	3.0 (0.7 - 68.8)	3.0 (0.9 - 313.0)
**TNM stage** (I/IIA/IIB/III/IV)	48/78/133/0/0	0/0/0/130/0	0/0/0/0/132	54/63/156/0/0
**Tumour size** (cm, mean ± *SD*)	4.65 ± 1.75	/	/	4.49 ± 1.38
**Lymph node metastasis** (yes/no)	133/126	/	/	156/117
**Differentiation** (well, moderate/poor)	167/92	/	/	177/96
**Neural invasion** (yes/no)	218/41	/	/	185/88
**Microvascular invasion** (yes/no)	64/195	/	/	83/190
**Chemotherapy** (yes/no)	210/49	114/16	122/10	186/87
**Chemoradiotherapy** (yes/no)	66/193	29/101	16/116	39/234

All patient clinicopathological and outcome data were registered in the pancreatic cancer database of the two institutions, as previously described [7, 10, 15]. This study was reviewed and approved by the Human Research Ethics Committee of Shanghai Cancer Center and Shanghai Huashan Hospital. Informed consent was obtained from each patient according to the committee's guidelines.

### Patient follow-up

A detailed description of the follow-up protocol was reported in our previous study [7, 10, 15, 16]. All patients were routinely monitored using clinical and laboratory examinations, which included measurement of CA19-9 serum levels, every three months until March 2015. Imaging examinations, including computed tomography (CT), magnetic resonance imaging, bone scan, or positron emission tomographic scanning (PET/CT), were also selectively performed. Overall survival (OS) was defined as the interval from the time of initial cytological or histological diagnosis to the date of death or last follow-up. For patients who underwent radical pancreatectomy, recurrence-free survival (RFS) was defined as the interval between surgery and tumor recurrence; if recurrence was not diagnosed, the RFS period ended on the date of death or the last follow-up. Postoperative recurrence was divided into two categories: local recurrence and distant metastasis [17]. Local recurrence was defined as appearing in the retroperitoneal area, including in the resection bed, remnant pancreas, or regional nodes. Distant metastasis was defined as any recurrence that occurred in distant organs, including the liver, lung, and bone. Radiographic findings consistent with recurrent disease were considered adequate proof of recurrence. Pathological assessment, consisting of histological or cytological evidence from the local recurrence or metastatic deposits, was rarely performed. All diagnostic determinations of pancreatic cancer recurrence involved multidisciplinary consultation. “Early” recurrence was defined as relapse within six months of surgery.

### Serum tumor marker measurement

The levels of the eight serum indexes used in this study were determined using radioimmunoassay kits manufactured by Abbott Laboratories (Chicago, IL, USA). Detailed information about the timing of blood draws (pretreatment or postoperative) is shown in Supplementary Materials and Methods.

### Pancreatic cancer staging

Pancreatic cancer stage was classified according to the AJCC TNM Staging of Pancreatic Cancer (7^th^ Edition, 2010). [14] Staging and included both clinical and pathological assessments as prescribed by the NCCN guidelines [18]. Detailed information is provided in Supplementary Materials and Methods. Additionally, pathological extension of pancreatic cancer though hepatic and lymph node metastasis was classified. Classification of lymph node metastasis was performed using resection specimens. Patients were divided into three groups based on the number of positive lymph nodes: Group I (no positive nodes), Group II (1–3 nodes), and Group III (> 3 nodes). H-classification defined the extent of hepatic metastasis in patients with stage IV disease. H1 represented liver metastasis with fewer than 5 nodules smaller than 3cm, H3 indicated more than 3 nodules larger than 3 cm, and H2 was defined as metastasis that did not classify as H1 or H3. These classifications were performed by radiologists and pathologists without knowledge of the experimental purpose.

### Statistical analyses

The predictive accuracy index was assessed by area under the receiver operating characteristic (ROC) curve analyses. To best preoperatively predict early metastasis in distant organs following surgery, the optimal cut-off point for high versus low serum CA125 levels was selected by ROC curves in the training cohort and was confirmed in the validation cohort, without re-estimation [19]. This cut-off point was the point at which the optimal sensitivity and specificity were achieved to yield the minimum value in the equation (1-sensitivity)^2^ + (1-specificity)^2^. Qualitative variables were analyzed by Pearson's χ2 or Fisher's exact test, and quantitative variables were analyzed using Student's *t*-test, the Mann-Whitney U test, or the Kruskal-Wallis test. Kaplan-Meier analyses were used to determine OS and RFS. Patient survival among subgroups was compared using the log-rank test. The Cox regression model was used for multivariate analyses. All statistical analyses were performed with SPSS 13.0 for Windows (SPSS, Chicago, IL, USA), and *p*<0.05 (two-tailed) was considered statistically significant. Unsupervised hierarchical clustering analysis was performed with Cluster 3.0 (Stanford University) using average linkage algorithms. The results of clustering were visualized using TreeView (Stanford University).

## SUPPLEMENTARY MATERIALS AND METHODS, FIGURES AND TABLES


