# Supplementary material for: Serum CA125 is a novel predictive marker for pancreatic cancer metastasis and correlates with the metastasis-associated burden
Source: Oncotarget. 2016 Jan 5;7(5):5943–56. doi: 10.18632/oncotarget.6819 (PMC4868732; doi:10.18632/oncotarget.6819)
Supplement: Supplementary file 1 [file oncotarget-07-5943-s001.pdf]

SUPPLEMENTARY MATERIALS AND METHODS

Serum tumor marker measurement (details)

In this study, the levels of eight serum indexes were determined using radioimmunoassay kits manufactured by Abbott Laboratories (Chicago, IL, USA). Baseline levels of these markers were defined as the last available measurement prior to resection or non-surgical treatment. The mean time of determination of these tumor marker levels before pancreatectomy (Stage I–II disease) was 5.9 ± 3.1 days in the training cohort from our institution (Shanghai Cancer Center) and 6.0 ± 3.3 days in the validation cohort from Shanghai Huashan Hospital. In our institution, the mean time of measurement of tumor marker levels before non-surgical treatment was 5.6 ± 2.6 days in patients with locally advanced disease (Stage III) and 5.4 ± 2.5 days in patients with metastatic disease (Stage IV). All indexes were detected at a similar time before treatment ($P = 0.372$, Kruskal-Wallis Test), thus avoiding any potential bias. Postoperative levels of serum biomarkers were measured three months after surgery.

Pancreatic cancer staging (details)

The classification criteria of pancreatic cancer staging in this study followed the AJCC TNM Staging of Pancreatic Cancer (7th Edition, 2010).[1] Staging included both clinical and pathological assessments as prescribed by the NCCN guidelines.[2] Clinical staging of primary tumors or metastasis was determined by an experienced surgeon and radiologists, without knowledge of the study. Staging primarily required high-quality, multiphase imaging implemented by contrast-enhanced pancreatic protocol CT and/or MRI. Additional imaging modalities, including endoscopic ultrasound, endoscopic retrograde cholangiopancreatography, PET/CT scans, and laparoscopy, were implemented in the case of uncertain findings obtained by standard imaging. All clinical evaluations were verified by pathological assessment, which consisted of histological or cytological evidence from the primary tumors or metastatic deposits. Independent pathologists who had no knowledge of the study determined pathological characteristics of the cancer, primary tumor staging (T1, T2 and T3), lymph node staging (N0 and N1), number of lymph nodes retrieved, number of positive lymph nodes, pathological type, histological grade, resection margins, neural invasion, and vascular invasion.

Cell lines and immunoblot analyses

HEK-293T cells and six human pancreatic cancer cell lines with different metastatic potential were purchased from American Type Culture Collection (ATCC, Rockville, MD). A previously described lentivirus-mediated transfection method was used to produce HEK-293T cells with Flag-CA125 fusion protein.[3, 4] Whole cell extracts from pancreatic cancer cells and FLAG-tagged CA125 expressing HEK-293T cells were isolated as described previously.[4, 5] Conditioned medium from the above cells was collected and concentrated using YM-10 MW Centricon filters (Millipore, Bedford, MA). Immunoblotting was performed as described previously.[6] Antibodies against FLAG, CA125, and β-actin were purchased from Abcam Ltd. (Cambridge, UK).

Real-time polymerase chain reaction and immunostaining analyses

mRNA expression of 17 metastasis-associated genes in RNAlater (Qiagen)-protected tumor samples from 49 patients with pancreatic cancer who received radical pancreatectomy were evaluated by real-time reverse transcription-polymerase chain reaction as described in a previous study.[6] Primer sequences used for amplification of selected genes and *GAPDH* are described in Table S4. Relative expression of the selected genes was quantified by normalization against *GAPDH* according to the $2^{-\Delta\Delta CT}$ method. Unsupervised hierarchical analysis was used to organize patients and metastasis-associated genes in a tree structure according to their similarities. The relationship between patients and genes was described graphically in a dendrogram, and the length of the branches represented the degrees of correlation between patients and genes. Unsupervised hierarchical clustering analyses were performed with Cluster 3.0 (Stanford University) using average linkage algorithms according to the instructions using dedicated software (http://bonsai.hgc.jp/~mdehoon/software/cluster/cluster3.pdf). The results of clustering were visualized by TreeView (Stanford University).

Tissue microarrays (TMAs) containing paraffin-embedded tumor samples from 107 patients in the training cohort were constructed as previously described.[3, 6, 7] The immunostaining assays for CA125, KRAS, CDKN2A/p16, TP53, and SMAD4/DPC4 were performed as previously described.[3, 4, 6] Additional matched samples of metastatic lymph nodes and primary tumors from 56 patients with Stage IIb pancreatic cancer and matched samples of metastases in liver and primary tumors from 16 patients with Stage IV pancreatic cancer were analyzed for CA125 expression. Primary antibodies used in this study were mouse anti-human CA125 antibody (1:50; Dako); mouse anti-human KRAS antibody (1:100; Abcam); rabbit anti-human CDKN2A/p16 (1:200; Abcam); mouse anti-human TP53 antibody

(1:100; Abcam); and mouse anti-human SMAD4/DPC4 antibody (1:100; Santa Cruz Biotechnology). Negative controls were processed without primary antibodies. Grading of CA125 staining intensity in tumor cells was classified as follows: 0 (no staining), 1 (weak expression), 2 (moderate expression), and 3 (strong expression). The percentage of CA125-positive stained tumor cells was graded as 0 (no staining), 1 (1%–50%), or 2 (51%–100%). The total CA125 score was calculated by the sum of the intensity and percentage of CA125 staining, and was used to define tissues as positive (score: 3–5) or negative (score: 0–2) for CA125 staining. Positive expression of KRAS, CDKN2A/p16, TP53, and SMAD4/DPC4 in tumors was scored as presence of immunostaining in ≥ 5% of tumor cells. Immunostaining was assessed independently by two observers without knowledge of the study. A consensus was achieved to resolve any discrepancies.

SUPPLEMENTARY RESULTS

Patient characteristics

Table 1 displays detailed clinicopathological characteristics for a total of 794 patients with pancreatic cancer from two independent high-volume centers. Of the 259 patients with stage I/II disease who underwent pancreatectomy at our institution (Shanghai Cancer Center), 175 patients experienced postoperative recurrence. These included 71 (40.6%) patients with local recurrence, 78 (44.6%) patients with distant metastasis, and 26 (14.8%) patients with synchronous distant and local recurrence. The median OS and RFS times of patients who underwent pancreatectomy were 17.7 months and 9.8 months, respectively. The 1- and 3-year OS rates were 64.8% and 23.6%, respectively, and the 1- and 3-year RFS rates were 42.2% and 20.5%, respectively. Two additional subgroups of patients with stage III and stage IV disease, collected from Shanghai Cancer center, had median OS times of 9.2 months (stage III) and 5.5 months (stage IV). The 1- and 2-year OS rates were 34.2% and 8.7%, respectively, for stage III disease, and 22.3% and 7.0%, respectively, for stage IV disease. These data of the patient population conformed to international trends. Similarly, of the additional 273 independent patients with stage I/II disease who underwent pancreatectomy at Shanghai Huashan Hospital, 196 patients experienced postoperative recurrence, including 33.7% (66/196) with local recurrence, 48.4% (95/196) with distant metastases, and 17.9% (35/196) with both distant and local recurrence. The median OS and RFS of the total group of resected patients were 16.2 months and 10.6 months, respectively. The 1-, 3-, and 5-year OS rates were 64.0%, 25.7%, and 19.6%, respectively, and the 1-, 3-, and 5-year RFS rates were 43.7%, 22.8%, and 17.5%, respectively.

Source of serum CA125 in patients with pancreatic cancer

Tissue microarray analysis of 107 pancreatic cancer patients (Stage I/II) showed that patients with positive CA125 staining had significantly higher serum CA125 levels than those with negative staining ($P<0.001$; Figure S2A). Immunoblot analysis of HEK-293T cells transfected with FLAG-CA125 fusion protein revealed FLAG-tagged CA125 protein in both the whole cell lysate and in conditioned media (Figure S2B). The increase in CA125 expression in pancreatic cancer cells was paralleled by an increased concentration of CA125 in their conditioned medium. A significant association between CA125 levels and metastatic potential in these cells was observed (Figure S2B). Immunostaining of clinical pancreatic cancer samples showed that CA125 was mainly present on the membrane of tumor cells. No staining was detected in stromal cells or normal pancreas cells (Figure S2C). In the paired specimens described in SUPPLEMENTARY MATERIALS AND METHODS, CA125 expression was predominantly observed on the membranes of metastatic cancer cells, but was not detected in the surrounding lymph nodes or liver cells (Figure S2D). The rate of positive CA125 expression in lymph node metastatic foci was 80.4% (45/56), which was significantly higher than that in the matched primary tumors [42.9% (24/56); $P<0.001$]; moreover, the rate of CA125 staining was higher in liver metastatic foci [87.5% (14/16)] than in matched primary tumors [43.8% (7/16); $P=0.009$)]. This evidence indicates that serum CA125 in patients mainly originated from pancreatic cancer cells exhibiting metastatic characteristics.

SUPPLEMENTARY REFERENCES

1. Edge SBB DR, Compton CC, Fritz AG et al. AJCC Cancer Staging Manual (ed 7th Edition). New York: Springer 2010.
2. The NCCN Clinical Practice Guidelines in Oncology (NCCN Guidelines™). The most recent and complete version of the NCCN Guidelines™ was available at: NCCN.org.
3. Liu L, Zhu XD, Wang WQ et al. Activation of beta-catenin by hypoxia in hepatocellular carcinoma contributes to enhanced metastatic potential and poor prognosis. *Clin Cancer Res.* 2010;16:2740-2750.
4. Liu L, Xu HX, Wang WQ et al. Cavin-1 is essential for the tumor-promoting effect of caveolin-1 and enhances

its prognostic potency in pancreatic cancer. *Oncogene*. 2014;33:2728-2736.

5. Liu L, Ren ZG, Shen Y et al. Influence of hepatic artery occlusion on tumor growth and metastatic potential in a human orthotopic hepatoma nude mouse model: relevance of epithelial-mesenchymal transition. *Cancer Sci*. 2010;101:120-128.
6. Xu HX, Zhu XD, Zhuang PY et al. Expression and prognostic significance of placental growth factor in hepatocellular carcinoma and peritumoral liver tissue. *Int J Cancer*. 2011;128:1559-1569.
7. Xu HX, Chen T, Wang WQ et al. Metabolic tumour burden assessed by F-FDG PET/CT associated with serum CA19-9 predicts pancreatic cancer outcome after resection. *Eur J Nucl Med Mol Imaging*. 2014;41:1093-1102.

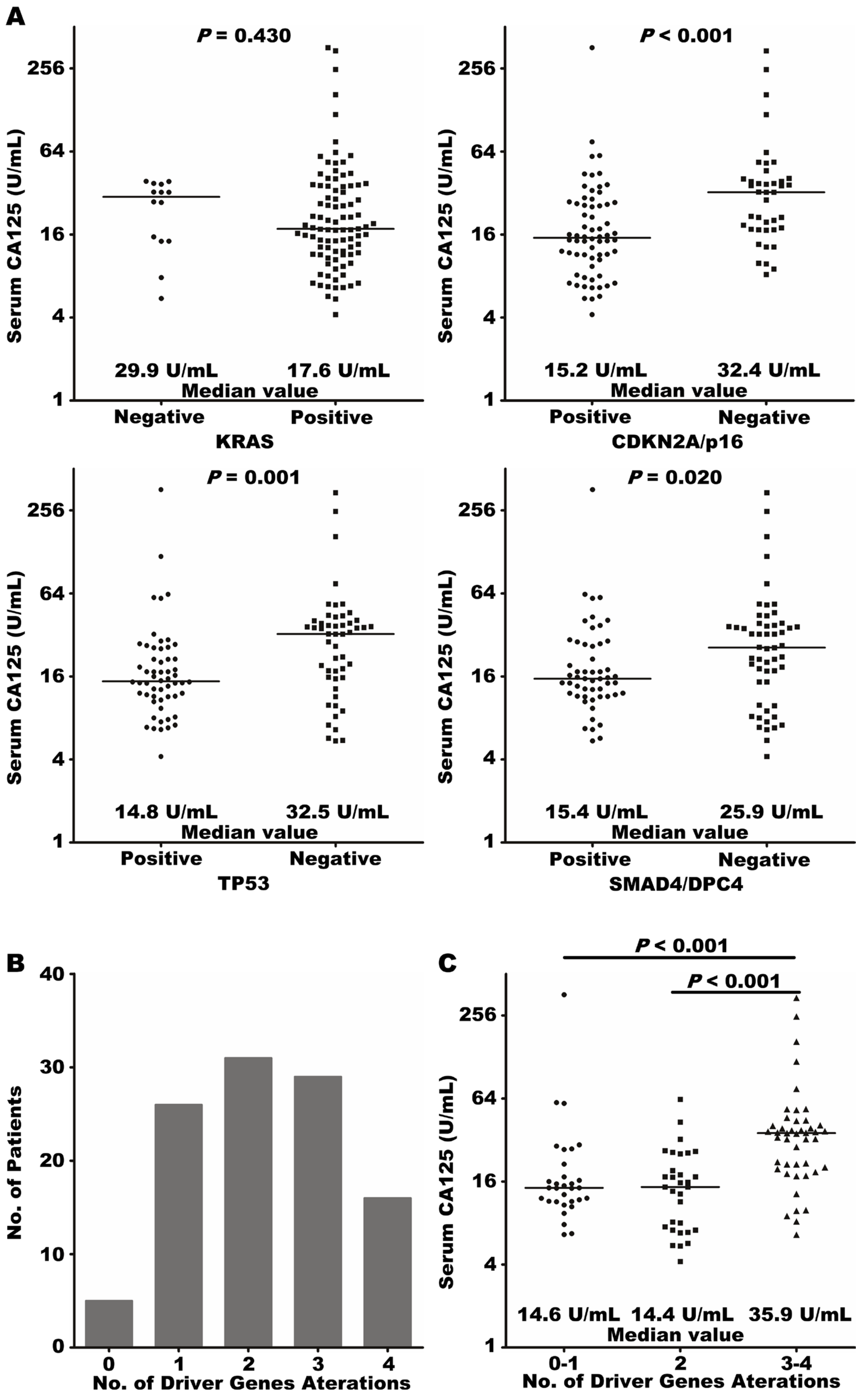

Supplementary Figure S1: A. Levels of baseline serum CA125 in patients with positive KRAS expression, and in patients with negative expression of CDKN2A/p16, TP53, or SMAD4/DPC4 proteins. **B.** The prevalence of combinations of alterations in these four genetic driver genes is depicted in a column bar graph. **C.** Levels of baseline serum CA125 in patients with coexistence of 0–1, 2, or 3–4 altered driver genes. The lines across the dot plots indicate the median values.

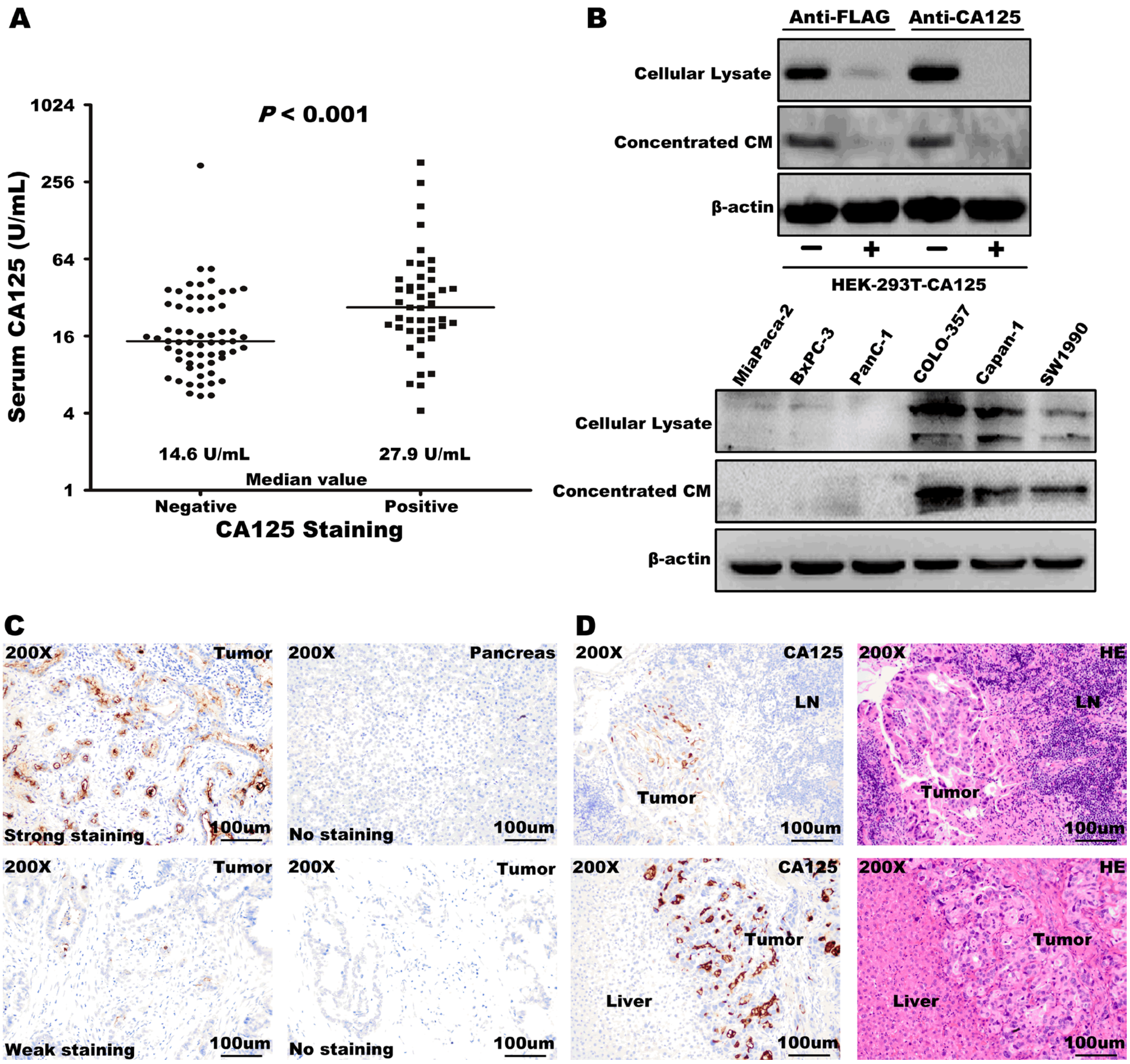

Supplementary Figure S2: A. Levels of baseline serum CA125 (log2 scale on the y-axis) in patients with positive/negative CA125 staining in primary tumors. The lines across the dot plots indicate the median values. **B.** FLAG-tagged CA125 protein was measured by immunoblotting in whole cell lysate and conditioned medium (CM) of HEK-293T cells transfected with or without FLAG-CA125 fusion protein. The expression of CA125 in pancreatic cancer cells and matched conditioned medium was measured by western blotting. **C.** Representative images of samples with strong, weak, and no CA125 staining in TMA analysis. **D.** Positive CA125 staining was observed in metastases to lymph nodes (LNs) or to liver.

Supplementary Table S1: ROC Curves Analysis for prediction of pancreatic cancer metastasis by serum tumor markers

Serum Tumor Markers	Metastasis Stage I *vs.* Stage IV			Metastasis Stage III *vs.* Stage IV			Lymph Node Metastasis Stage I/IIa *vs.* Stage IIb		
	AUC	95%CI	*P*	AUC	95%CI	*P*	AUC	95%CI	*P*
**Serum CA19-9 (U/mL)**	0.722	[0.648, 0.797]	< 0.001	0.588	[0.513, 0.663]	0.024	0.598	[0.529, 0.668]	0.006
**Serum CA125 (U/mL)**	0.892	[0.846, 0.936]	< 0.001	0.723	[0.657, 0.789]	< 0.001	0.693	[0.628, 0.758]	< 0.001
**Serum CEA (ng/mL)**	0.716	[0.637, 0.796]	< 0.001	0.590	[0.516, 0.664]	0.021	0.570	[0.501, 0.640]	0.050
**Serum CA242 (U/mL)**	0.688	[0.607, 0.769]	< 0.001	0.583	[0.507, 0.658]	0.034	0.619	[0.550, 0.688]	0.001
**Serum CA50 (U/mL)**	0.554	[0.465, 0.643]	0.270	0.559	[0.484, 0.635]	0.128	0.589	[0.520, 0.658]	0.013
**Serum CA72-4 (U/mL)**	0.673	[0.592, 0.754]	< 0.001	0.605	[0.531, 0.678]	0.007	0.511	[0.441, 0.582]	0.752
**Serum CA153 (U/mL)**	0.667	[0.585, 0.749]	0.001	0.577	[0.502, 0.652]	0.050	0.591	[0.522, 0.660]	0.011
**Serum AFP (ng/mL)**	0.541	[0.446, 0.636]	0.398	0.508	[0.429, 0.586]	0.844	0.521	[0.450, 0.591]	0.566

Supplementary Table S2: Comparison among serum tumor markers for prediction of pancreatic cancer metastasis by ROC curves analysis

Comparison	Metastasis			Metastasis			Lymph Node Metastasis		
	Stage I *vs.* Stage IV			Stage III *vs.* Stage IV			Stage I/IIa *vs.* Stage IIb		
	Difference between AUC	95%CI	*P*	Difference between AUC	95%CI	*P*	Difference between AUC	95%CI	*P*
**CA125 *vs.* CA19-9**	0.170	[0.084, 0.255]	< 0.001	0.135	[0.045, 0.224]	0.003	0.094	[0.013, 0.176]	0.023
**CA125 *vs.* CEA**	0.176	[0.088, 0.263]	< 0.001	0.133	[0.055, 0.211]	< 0.001	0.122	[0.044, 0.201]	0.002
**CA125 *vs.* CA242**	0.204	[0.112, 0.295]	< 0.001	0.140	[0.050, 0.230]	0.002	0.074	[-0.009, 0.157]	0.084
**CA125 *vs.* CA50**	0.338	[0.239, 0.437]	< 0.001	0.163	[0.068, 0.259]	< 0.001	0.104	[0.021, 0.187]	0.015
**CA125 *vs.* CA72-4**	0.219	[0.131, 0.307]	< 0.001	0.118	[0.027, 0.209]	0.011	0.181	[0.082, 0.281]	< 0.001
**CA125 *vs.* CA153**	0.225	[0.143, 0.306]	< 0.001	0.146	[0.067, 0.225]	< 0.001	0.102	[0.021, 0.183]	0.013
**CA125 *vs.* AFP**	0.351	[0.255, 0.446]	< 0.001	0.215	[0.120, 0.310]	< 0.001	0.172	[0.078, 0.266]	< 0.001
**CA19-9 *vs.* CEA**	0.006	[-0.091, 0.103]	0.905	0.002	[-0.089, 0.092]	0.973	0.028	[-0.060, 0.116]	0.533
**CA19-9 *vs.* CA242**	0.034	[-0.005, 0.073]	0.086	0.006	[-0.034, 0.045]	0.782	0.021	[-0.015, 0.056]	0.257
**CA19-9 *vs.* CA50**	0.169	[0.094, 0.243]	< 0.001	0.029	[-0.026, 0.084]	0.302	0.009	[-0.045, 0.063]	0.739
**CA19-9 *vs.* CA72-4**	0.050	[-0.054, 0.153]	0.349	0.016	[-0.082, 0.115]	0.745	0.087	[-0.015, 0.189]	0.094
**CA19-9 *vs.* CA153**	0.055	[-0.050, 0.160]	0.303	0.012	[-0.087, 0.111]	0.818	0.007	[-0.084, 0.099]	0.872
**CA19-9 *vs.* AFP**	0.181	[0.059, 0.304]	0.004	0.081	[-0.021, 0.183]	0.121	0.078	[-0.023, 0.179]	0.131

Supplementary Table S3: Clinicopathological features of patients with pancreatic cancer after radical resection in the prospective cohort of shanghai cancer center and the extended cohort of shanghai huashan hospital

Features	Extended data<br>Shanghai Huashan hospital<br>***n = 384***	Prospective data<br>Shanghai Cancer Center<br>***n = 142***
**Age** [years, median (range)]	61 (20 - 79)	62 (35 - 81)
**Gender** (male/female)	237/147	85/57
**Tumour location** (head/body, tail)	314/70	142/0
**Serum CA19-9** [U/mL, median (range)]	136.3 (0.6 - 20740.0)	137.3 (0.6 - 15461.0)
**Serum CA125** [U/mL, median (range)]	25.1 (2.1 - 666.5)	19.9 (4.3 – 280.5)
**TNM stage** (I/IIA/IIB)	82/76/226	32/42/68
**Tumour size** (cm, mean ± *SD*)	4.40 ± 1.33	3.29 ± 1.17
**Lymph node metastasis** (yes/no)	158/226	68/74
**Differentiation** (well, moderate/poor)	250/134	85/57
**Neural invasion** (yes/no)	255/129	122/20
**Microvascular invasion** (yes/no)	117/267	30/112
**Chemotherapy** (yes/no)	261/123	135/7
**Chemoradiotherapy** (yes/no)	56/328	28/114

Supplementary Table S4: List of primer sequences

Gene	Gene name	Primer	Oligonucleotide sequence
**SNRPF**	Small nuclear ribonucleoprotein F	Forward	5'-GTAGCCTGCAACATTCGGC-3'
		Reverse	5'-CCCTTGTACTCCATTCCCCAC-3'
**EIF4EL3**	Elongation initiation factor 4E-like 3	Forward	5'-ACAACAAGTTCGACGCTTTGA-3'
		Reverse	5'-TCTCTTGCTACTGCTCTGATTCT-3'
**HNRPAB**	Heterogeneous nuclear ribonucleoprotein A/B	Forward	5'-ATTGAGGCCATTGAATTGCCA-3'
		Reverse	5'-GGCCACCTTGATCTCACACTT-3'
**DHPS**	Deoxyhypusine synthase	Forward	5'-TACTTGGGCGAGTTTAGCCTC-3'
		Reverse	5'-GTCCACTTTACACCCTCTGTG-3'
**PTTG1**	Securin	Forward	5'-ACCCGTGTGGTTGCTAAGG-3'
		Reverse	5'-ACGTGGTGTTGAAACTTGAGAT-3'
**COL1A1**	Type 1 collagen, α1	Forward	5'-GAGGGCCAAGACGAAGACATC-3'
		Reverse	5'-CAGATCACGTCATCGCACAAC-3'
**COL1A2**	Type 1 collagen, α2	Forward	5'-GTTGCTGCTTGCAGTAACCTT-3'
		Reverse	5'-AGGGCCAAGTCCAACTCCTT-3'
**LMNB1**	Lamin B1	Forward	5'-ACATGGAAATCAGTGCTTACAGG-3'
		Reverse	5'-GGGATACTGTCACACGGGA-3'
**ACTG2**	Actin, γ2	Forward	5'-GCGTGTAGCACCTGAAGAG-3'
		Reverse	5'-GAATGGCGACGTACATGGCA-3'
**MYLK**	Myosin light chain kinase	Forward	5'-CACCGTCCATGAAAAGAAGAGTAG-3'
		Reverse	5'-GAGAGGCCCTGCAGGAAGATGG-3'
**MYH11**	Myosin, heavy chain 11	Forward	5'-CATCTACTCGGAGAAGATCGTCG-3'
		Reverse	5'-CGCCTGTGCATAGAATGGACT-3'
**CNN1**	Calponin 1	Forward	5'-CTGTCAGCCGAGGTTAAGAAC-3'
		Reverse	5'-GAGGCCGTCCATGAAGTTGTT-3'
**HLA-DPB1**	MHC Class II, DPβ1	Forward	5'-ATTCTGCCCGGAGTAAGACAT-3'
		Reverse	5'-TCGTTGAACTTTCTTGCTCCTC-3'
**RUNX1**	Runt-related transcription factor 1	Forward	5'-CTGCCCATCGCTTTCAAGGT-3'
		Reverse	5'-GCCGAGTAGTTTTCATCATTGCC-3'
**MT3**	Metallothionein 3	Forward	5'-GACCTGCCCCTGCCCTTCTGGTGG-3'
		Reverse	5'-GCTCCACACGGAGGGGTGCCTTCT-3'
**NR4A1**	Nuclear hormone receptor TR3	Forward	5'-CTGCCAATCTCCTCACTTCC-3'
		Reverse	5'-CAGCATCTTCCTTCCCAAAG-3'
**RBM5**	RNA binding motif 5	Forward	5'-CCATCACAGAGAGCGATATTCG-3'
		Reverse	5'-CGGCTTACACCTGTTTTCCTC-3'
**GAPDH**	Glyceraldehyde-3-phosphate dehydrogenase	Forward	5'-GGAGCGAGATCCCTCCAAAAT-3'
		Reverse	5'-GGCTGTTGTCATACTTCTCATGG-3'
